# A simple framework to distinguish ‘individualistic’ from a ‘uniform rate’ of ageing within or between study populations

**DOI:** 10.1007/s11357-023-00866-7

**Published:** 2023-07-11

**Authors:** Hans Degens, Gladys L. Onambélé-Pearson

**Affiliations:** 1 Department of Life Sciences, Research Centre for Musculoskeletal Science & Sports Medicine, Institute of Sport, Manchester Metropolitan University, Manchester, UK; 2 Institute of Sport Science and Innovations, Lithuanian Sports University, Kaunas, Lithuania; 3 Department of Sport and Exercise Science, Research Centre for Musculoskeletal Science & Sports Medicine, Institute of Sport, Manchester Metropolitan University, Manchester, UK

**Keywords:** Ageing, Individualistic, Uniform, Rate

## Abstract

Ageing is accompanied by a progressive decline in physiological functions. It is often argued that the rate of ageing differs between people and is ‘highly individualistic’. This view is not unequivocally shared, and others have argued that the rate of ageing is rather ‘uniform’. Distinguishing conclusively between these views requires longitudinal data, but these are difficult to obtain as they require decades of data collection from individuals. Here, a simple framework is proposed to assess in cross-sectional data whether in a given population the rate is ‘highly individualistic’ or rather ‘uniform’. It is illustrated that an age-related decrease in the standard deviation (SD) of a certain parameter combined with a non-changing coefficient of variation (COVAR) reflects a ‘uniform’ rate of ageing, whilst an increase or decrease in COVAR with or without a concomitant increase in SD reflects a ‘highly individualistic’ rate of ageing. This framework is applied to some published data, focussing on muscle strength, power and physical function for the sake of illustration, and it is suggested that most studies do in fact show a ‘highly individualistic’ rate of ageing, perhaps apart from a ‘uniform’ rate of ageing in master athletes.

## Introduction

Ageing is accompanied by a progressive decline in the function of many, if not all, physiological systems, ultimately resulting in the demise of an organism. The rate of decline seems virtually identical in a range of physiological systems [1], also reflected by a constant ratio of maximal aerobic:maximal anaerobic power, at least in master cyclists [2]. This suggests that ageing affects all physiological systems in a similar way, further illustrated by the largely similar age-related rate of decline in performance between many athletic disciplines, both in cross-sectional and longitudinal data [3]. There is, however, the question whether there is a ‘uniform’ rate of ageing within a population [4] or whether the rate of ageing is ‘highly individualistic’ [5].

Distinguishing between the two positions is rather difficult. Nevertheless, this question has been given some thought in the literature. For instance, the argument for a ‘uniform rate of ageing’ was that the variation in for instance muscle mass or strength in the older population is just a reflection of the variation this population would have exhibited when they were young [4]. In support of this, it was found not only by our group [6] but also by others [7] that the standard deviation (SD) in muscle mass, strength, power and physical performance stays essentially constant during ageing, which was also the case for the SD in the maximal oxygen consumption (VO_2_max) of 55- to 79-year-old active cyclists [5].

The latter authors argued, however, that physical activity will modify the rate of ageing, as it is well known that regular endurance or resistance training can increase muscle mass and strength, or endurance and VO_2_max, respectively. In support of this, master endurance athletes and power athletes have a higher VO_2_max [8] and muscle power [9] than their age-matched counterparts. This suggests that besides modulation of the rate ageing by other factors such as smoking, lifestyle and genetics [10–13], physical activity does indeed appear to reduce the rate of ageing.

There is no doubt that exercise can improve VO_2_max and muscle strength, even when one starts exercise later in life. However, the absolute rate of decline in VO_2_max or strength is larger in athletes than non-athletes [8, 9], which suggests that the rate of ageing is faster, rather than slower, in athletes compared to non-athletes. On the other hand, the annual percentage decline is similar in athletes and non-athletes, resulting in a converging of the lines of decline with increasing age [14–16]. It is therefore suggested that the inherent ageing process is not attenuated, but rather that any better function in an athlete than an age-matched non-athlete is attributable to capitalising on the ability to adapt to altered functional demands.

Finally, it is often argued that master athletes are a self-selected population of high performers, and one would expect that those that continue master athletics after the age of 80 will have a better performance at the age of 80 than those that stopped master athletics at 80 years. Yet, our group previously observed that their performance was similar [17], indicating that it is not because of poor performance that these 80-year-old athletes stopped competing. In addition, the coefficient of variation in performance remained rather constant, further suggesting a ‘uniform rate of ageing’.

**Fig. 1:**
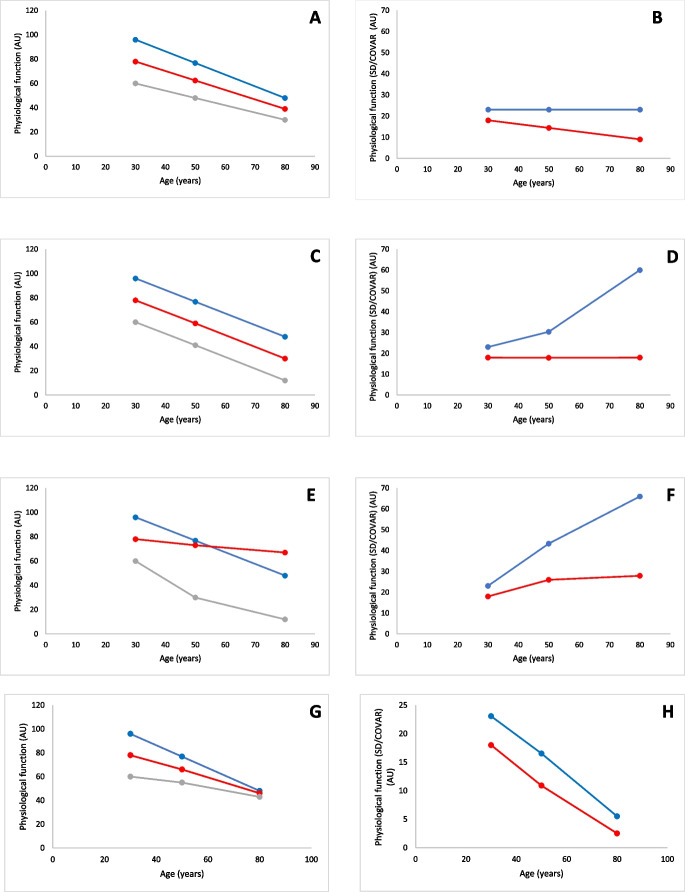
The three lines in **A, C, E,** and **G** represent three different individuals. The value on the y-axis represents any physiological function (e.g. muscle power, VO_2_max, or cognitive function). In **B, D, F,** and **H**, the red line indicates the standard deviation (SD) and the blue line the coefficient of variation (COVAR). **A** Each person loses 1% of their physiological function each year so that at the age of 80 years only 50% of the original physiological function is left. In this situation, the **B** SD of this population of 3 people decreases with age, whilst the COVAR is constant. **C** Each person loses an identical absolute amount of the physiological function each year. In this situation, the **D** SD stays constant, but the COVAR increases with ageing. **E** In this case, there is just a random decrement in physiological function with age with one showing a rapid decline, another a modest decline, and the third barely any decline at all and here **F** both the SD and COVAR increase. **G** The person with the highest starting value loses the largest absolute amount, and the person with the lowest starting value loses the smallest absolute amount of the physiological function each year, and the individual in between loses an amount of physical function each year that is between the other two. In this case **H** both the SD and COVAR decrease over time

The above discussion shows that there are as good arguments to believe that the rate of ageing is ‘highly individualistic’ as there are to support that it is ‘uniform’. The problem is how to distinguish between the two? One obvious way is to assess in many individuals the changes in a given physiological function over the lifespan. Given that such studies require data collection over decades, we suggest here a framework to distinguish a ‘highly individualistic’ or ‘uniform’ rate of ageing in a study population (Fig. 1).

## Proposed framework

Consider three hypothetical individuals (Fig. 1A, C, E, G), all tracked through an extended number of years, and each with a different physiological function (e.g. muscle power, VO_2_max, or cognitive function) at the age of 30. They are ranked as having best, worst and intermediate physiological function. Next, consider also the possible variability in standard deviation (SD i.e. a measure of data dispersion in relation to the average value, where a low standard deviation occurs when data is clustered around the mean, and high standard deviation occurs when data is more scattered) (Fig. 1B, D, F) and the coefficient of variation (COVAR: 100%*(SD/average); i.e. the ratio of the standard deviation to the mean). The smaller the COVAR, the lower the level of dispersion around the mean of the physiological functions of interest, from age 30 through to age 80.

### Similar relative rate of decline

In Fig. 1A, we assume that each person loses 1% of their physiological function at the age of 30 each year so that in each individual at the age of 80 years only 50% of the original physiological function is left. With increasing age, the absolute difference in physiological function between the three individuals becomes smaller, something that is also seen in athletes compared to non-athletes. In this scenario, the SD decreases with age, whilst the COVAR remains constant (Fig. 1B), and the regression lines of the annual % decrease have identical slopes (regression line not shown).

### Similar absolute rate of decline

In a second scenario (Fig. 1C), the three hypothetical individuals have the same starting point as in scenario one above (Fig. 1A). However, in this case, each person loses an identical absolute amount of physiological function each year, as reflected by the parallel lines. In this case, the SD stays constant, but the COVAR increases with ageing (Fig. 1D).

### Random differences in rate of decline

In the third scenario (Fig. 1E), the three hypothetical individuals again have the same starting point as in scenarios 1 and 2 (Fig. 1A, C). However, in this case, there is just a random decrement in physiological function with age, with one individual showing a rapid decline, the second a modest decline, and the third barely any decline. In this case, both the SD and COVAR increase (Fig. 1F).

### Rate of decline inversely related to starting value

Finally, in the fourth scenario (Fig. 1G), the three hypothetical individuals again have identical starting points in the previous scenarios (Fig. 1A, C, E). However, in this scenario, the person with the highest starting physiological function value shows the steepest absolute and relative rate of decline, the person with the lowest physiological function starting value has the smallest absolute and relative rate of decline, and the person with an intermediate physiological function at the start shows a rate of loss of physical function between the other two. In this scenario, both the SD and COVAR decrease over time (Fig. 1H).

The first scenario is representative of a ‘uniform’ and the last scenario a ‘highly individualistic’ rate of ageing. The second scenario is somewhat in between, as in terms of the absolute rate the decrement is uniform, but in relative terms the person who was physiologically the worst off at age 30 is deteriorating faster than the person who was physiologically the most advantaged at age 30. Overall, we suggest that scenario 2 also reflects a ‘highly individualistic’ rate of ageing, as it is readily understandable that a taller person will have a larger muscle mass, strength, VO_2_max, cognitive function, etc. than a smaller stature individual, and representing ageing as a percentage decline from an original value takes this bias out of the equation.

In summary, these scenarios indicate that if there is no (significant) age-related change in COVAR for a physiological parameter of interest in a study population then that study population has a ‘uniform’ rate of ageing, and when both SD and COVAR change with age, we can conclude that even in absolute terms the rate is ‘highly individualistic’.

## Application

Both others and our group have previously shown that the SD in muscle mass, strength, power and physical function did not change significantly during ageing [6, 7]. The same applied to the VO_2_max of ageing cyclists [5] and it was argued that this indicated that the rate of ageing was ‘uniform’ rather than ‘highly individualistic’ [4]. Based on the current framework, where it reflects scenario 2, this stance is now changed, and it has to be concluded that indeed the ageing of the cyclists was ‘highly individualistic’, but perhaps not entirely as was suggested by the authors of the paper on ageing cyclists, who argued that physical activity modifies the ageing process, without providing comparison against a control group. We can in fact apply our framework to address the question whether exercise slows the rate of ageing or, in other words, whether ageing is uniform or non-uniform between athletes and non-athletes. As an example of the application of our framework, the absolute annual decline in power [9] or VO_2_max [8] is larger in athletes than non-athletes, but when expressed as % annual decline, the regression lines have similar slopes, indicating that the rates of ageing are similar in both populations, and hence, ageing is uniform between the two populations in these studies (scenario 1). A study on track-and-field master athletics in a variety of disciplines showed a progressive decrease in the SD and a rather constant COVAR during ageing [17], which implies—applying the proposed framework—that in track-and-field master athletes the rate of ageing is ‘uniform’ (scenario 1).

## Conclusion

The simple framework presented here can help researchers evaluate whether there is a ‘uniform’ or ‘highly individualistic’ rate of ageing in a study population by just assessing the age-related changes in the SD and COVAR. Application of this simple framework will perhaps also help to reveal any populations that indeed show a rather ‘highly individualistic’ rate of ageing, that in turn can help understand factors, such as environmental and behavioural factors, that reduce or accelerate the rate of ageing, crucial for the design of interventions and life-style changes that attenuate the ageing process and increase the number of healthy-life years.
